# Endoscopic Resection Techniques for Widespread Precancerous Lesions and Early Carcinomas in the Rectum

**DOI:** 10.3390/jcm14103322

**Published:** 2025-05-09

**Authors:** Juergen Hochberger, Martin Loss, Elena Kruse, Konstantinos Kouladouros

**Affiliations:** 1 Department of Medicine, Hepatology and Gastroenterology, Campus Virchow Klinikum, Charité University Medicine, 13353 Berlin, Germany; konstantinos.kouladouros@charite.de; 2 Department of Gastroenterology, Vivantes Klinikum im Friedrichshain, 10249 Berlin, Germany; 3 Medical Clinic I—Gastroenterology, Friedrich-Alexander University Erlangen-Nuremberg, 91054 Erlangen, Germany; 4 Department of General and Visceral Surgery, Vivantes Klinikum im Friedrichshain, 10249 Berlin, Germany; martin.loss@vivantes.de; 5 Department of Gastroenterology and Hepatology, Asklepios Westklinikum, 22559 Hamburg, Germany

**Keywords:** endoscopic submucosal dissection (ESD), endoscopic mucosal resection (EMR), endoscopic full-thickness resection (eFTR), endoscopic intermuscular dissection (EID), endoscopic en bloc resection, rectal cancer, T1 rectal cancer, endoscopic intervention

## Abstract

Today, endoscopy plays a crucial role not only in the detection of precancerous and malignant colorectal lesions, but also in the treatment of even widespread adenomas and T1 early cancers. In addition to classic polypectomy and endoscopic mucosal resection (EMR) using a snare, in recent years, endoscopic submucosal dissection (ESD) has become increasingly important. Marking, submucosal injection, circumferential incision of the mucosa around the lesion, tunneling, and submucosal dissection using a short diathermic knife facilitate the ‘en bloc’ resection of lesions larger than 3 cm, difficult to resect in one piece using a snare. Lesions with high-grade dysplasia or mucosal carcinoma are other good candidates aside from widespread adenomata with a high risk of recurrence after piecemeal resection. ESD allows R0 resection rates of more than 90% in specialized centers. Lesions of 20 cm have been removed ‘en bloc’ by expert endoscopists. ESD provides an optimal histopathologic yield and has a risk of recurrence as low as 3%. Endoscopic full-thickness resection using a special device (eFTRD) is another addition to the resection armamentarium. It is especially suitable for circumscribed lesions up to 2 cm in the middle and upper rectum. Endoscopic intermuscular dissection (EID) is a recent modification of ESD primarily in the rectum, including the inner, circular muscular layer into the resection specimen. In this way, it allows a histopathologic analysis of the entire submucosa beyond the mucosal and upper submucosal layer such as in ESD. This is especially important for T1 cancers invading the submucosa without any other risk factors of invasion.

## 1. Pre-Therapeutic Endoscopic Evaluation of the Lesion

**Figure 1:**
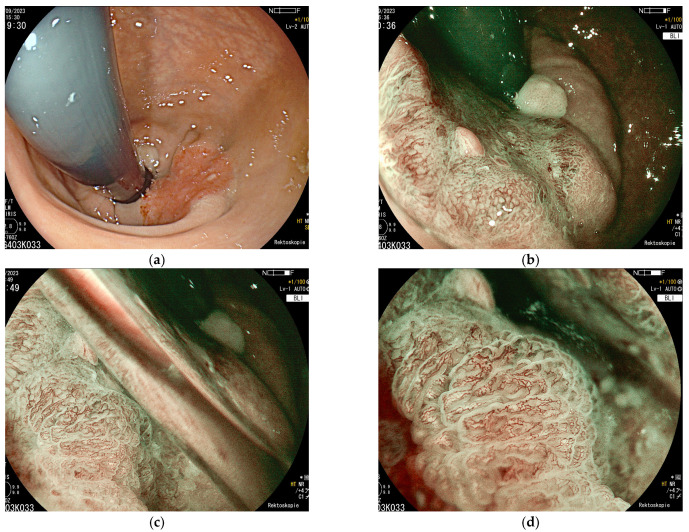
(**a**–**d**) Distal rectum in inversion: T1 early adenocarcinoma located at the ano-rectal verge towards the distal rectum in white light (**a**) and with digital chromoendoscopy (**b**–**d**); (blue light imaging, BLI; zoom magnification x3; EG760Z, Fujifilm, Tokyo, Japan). A pseudo-depressed surface structure (Paris IIa + c) and atypical vascular pattern (JNET classification 2b) can be seen.

Today, the colonoscopy is the most important tool for the early detection of colorectal cancer [1,2]. In recent years, the optical performance of flexible endoscopes has improved tremendously with an ultra-high image resolution, near focusing, and digital chromoendoscopy to highlight surface structures and vascular patterns (Figure 1a–d). The pre-treatment evaluation includes exact size and macroscopic appearance according to the Paris classification plus the Japanese classification for lateral spreading tumors [3,4,5,6]. The latter characterizes lesions larger than 2 cm according to the type of growth. In contrast to the Paris classification, the Japanese classification of laterally spreading tumors (LSTs) not only describes the type of growth but adds information on the endoscopic image of the mucosal surface. The LST classification discriminates between non-granular and granular surface patterns, polypoid, flat, or depressed areas, and, therefore, gives a much more detailed clinical information about the lesion compared to the Paris classification alone in clinical practice. In a prospective study of 2106 patients undergoing EMR for colorectal polyps, Burgess et al. analyzed the risk of occult neoplasia [7]. The risk factors were rectosigmoid location, non-granular surface structure, and Paris Classification 0-Is (polypoid, broad-based) or 0-IIa+Is (mixed flat and polypoid appearance). In contrast, flat granular lesions (Paris 0-IIa) showed a low risk of occult carcinoma [7].

The use of dyes sprayed via a catheter in a diluted solution on the tissue surface increases the visibility of the lesion and the demarcation towards the surrounding normal tissue, e.g., using indigocarmine blue 0.2%. Traditional dye-based chromoendoscopy can be imitated electronically using light of a specific light spectrum. This saves time and the specific light mode of the endoscope can be switched on and off by just pushing a button [8]. All modern endoscope systems allow an electronic switch to a blue-green light mode such as narrow band imaging (NBI) or blue light imaging (BLI) for a better characterization of the surface and vascular pattern (Figure 1a–d). Already, in 2007, Chiu et al. compared white light endoscopy, and low- and high-magnification NBI digital chromoendoscopy (CE), as well as conventional dye-based chromoendoscopy (DCE), using 0.2% indigocarmine blue sprayed on the lesion [9]. Pictures were taken of the 180 colorectal lesions in 133 patients. Subsequently, all lesions were resected endoscopically for histopathological analysis. Endoscopic images were stored electronically and randomly allocated to two readers for evaluation. The diagnostic accuracy of NBI with a low or high magnification was significantly higher than that of conventional colonoscopy (low magnification: *p* = 0.0434 for reader 1 and *p* = 0.004 for reader 2; high magnification: *p* = 0.001 for both readers) and was comparable to that of conventional chromoendoscopy [9].

The so-called Japanese Narrow-Band Imaging Expert Team (JNET) classification has been established in order to classify colonic lesions under blue-green light [10,11]. This includes features such as the loss of homogeneity of the surface structure, neovascularization, dilated vessels, and an unstructured pit pattern according to Kudo’s “pit pattern” classification considered as indicators of potentially infiltrating malignant growth. Figure 1a–d show a mucosal carcinoma of the distal rectum (Figure 1a–d).

The lifting of the lesion after a submucosal injection of saline is, today, only a relative criterion for the possibility of resection. If complete lifting occurs, resection can be performed safely in most cases. Non-lifting was, in former days, often a criterion for surgical resection. However, a “non-lifting sign” can also occur in submucosal fibrosis without malignancy [12,13]. It has been repeatedly shown that the assessment of the endoscopic resectability of a polyp is highly dependent on the experience of the endoscopist. The majority of potentially “unresectable” polyps can be removed successfully in expert centers [12]. It is not always possible to correctly predict the histology prior to resection. In particular, deep submucosal infiltration cannot always be adequately determined from the surface of the lesion. Unfortunately, endoscopic ultrasound (EUS) and MRI are often of little help in T1 carcinomas [12,14]. Detering et al. analyzed 5539 patients from the Dutch ColoRectal Audit database with surgical rectal resection and pT1 and pT2 cancers with regard to the accuracy of preoperative MRI and rectal EUS. We found 5288 patients had MRI only, and 251 MRI plus rectal EUS. Of the 834 patients with pT1N0 cancers, after surgical resection, only 253 (30.3%) had been staged correctly as cT1N0 preoperatively. The sensitivity and specificity of the combined MRI and ERUS was 69.0 and 72.6%, respectively, for T1 tumors only [14]. For rectal lesions with pre-interventional evidence of focal carcinoma in an adenoma and no evidence of deep infiltration, en bloc resection with clear margins should be attempted, e.g., as Endoscopic Intermuscular Dissection (EID) [15].

## 2. Endoscopic Resection Techniques

### 2.1. Endoscopic Mucosal Resection (EMR)

A local injection of saline underneath the lesion, and the subsequent resection using a diathermic snare, was described as saline-assisted mucosectomy as early as 1973 by Deyhle and colleagues [16]. For lesions larger than 2 cm, complete resection is usually only possible in several fractions (“piecemeal resection”). With the introduction of the later commonly called “endoscopic mucosal resection” (EMR) [17], the removal of flat and extensive lesions became possible. The advantage of EMR is that it is relatively easy to learn, takes less time, and has a lower complication rate compared to submucosal dissection (see below). However, the risk of local recurrence increases exponentially with the number of fragments relative to the size of the lesion (“Sidney resection coefficient”) [18]. This leads to recurrence rates of 15–40% for extensive polyps [19]. Tate, Bourke et al. from Australia compared the impact of the ‘en bloc’ (e-EMR) versus piecemeal resection of laterally spreading lesions 2 to 2.5 cm in size in 570 patients over a 10-year period [18]. e-EMR offered no additional advantage for predicted benign LSLs. However, it was associated with an increased risk of major deep mural injury. The rates of surgical referral were not significantly different between the groups at either surveillance interval [18]. Recurrence can be successfully reduced by coagulating the lateral margin of adjacent normal mucosa with the tip of the snare [20,21]. Endoscopic mucosal resection (EMR) as piecemeal resection can be an adequate procedure for flat, non-polypoid lesions with a low risk of malignancy [7]. EMR seems not adequate for polypoid lesions, which cannot be removed in one piece due to the increased risk of focal malignancy of those lesions larger than 2 cm in diameter [7]. Kouladouros et al. recently published a retrospective comparison on the outcome after endoscopic mucosal resection versus endoscopic submucosal dissection (ESD) for the treatment of rectal lesions involving the dentate line [22]. It was found that 68 patients/lesions were treated by ESD, compared to 62 patients/lesions in the EMR group. They found a significantly higher en bloc and histologically complete resection rate for the ESD group. ESD showed a significantly lower recurrence rate of 1.5% compared to EMR, with 25.8% recurrences during a median follow-up of 18 month in the ESD and 34 month in the EMR group. ESD showed a significant advantage in the curative resection of low-risk adenocarcinomas (G1,2; infiltration ≤ 2000 µm) of 62.5% in the ESD group vs. 14.3% in the EMR group (*p* = 0.033) [22].

### 2.2. Endoscopic Submucosal Dissection (ESD)

**Figure 2:**
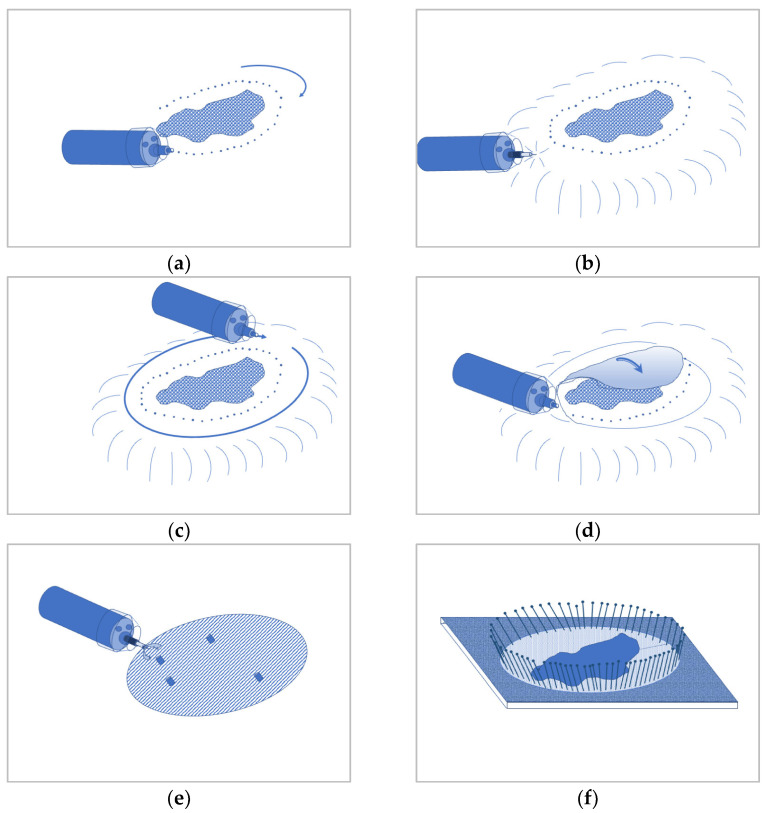
(**a**–**d**) Schematic illustration of the traditional steps of ESD: delineation and circumferential marking of the lesion plus a lateral safety margin using coagulation dots (**a**). Lifting of the resection area by submucosal injection (**b**). Circumferential incision with the electrosurgical knife (**c**), followed by submucosal dissection and tunneling underneath the lesion (**d**). After removal of the specimen vessels are clipped or coagulated at the resection ground (**e**). Circumferential pinning of the specimen cork before formalin fixation for proper evaluation of the rection margins (**f**). According to Hochberger et al. [24] with permission by the authors.

Endoscopic submucosal dissection (ESD) allows the ‘en bloc’ resection of even extensive mucosal lesions that cannot safely be resected in one piece with conventional snare resection. ESD even enables the resection of lesions with an underlying scar after a prior resection attempt or recurrence after surgical resection [12,13]. Figure 2 illustrates the different steps of the classic ESD procedure [23,24,25]. The endoscope is fitted with a cylindrical or conical transparent hood providing stability and optimizing vision during later incision and tissue dissection.

**Figure 3:**
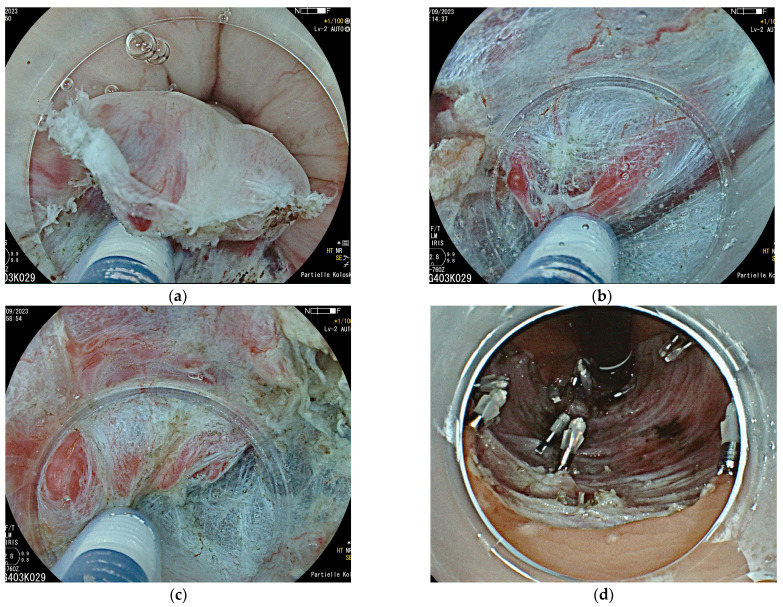
(**a**–**d**) Endoscopic submucosal dissection of the lesion in the distal rectum shown in Figure 1. After marking and submucosal fluid injection, an incision at the level of the upper anal channel is performed (**a**), with subsequent submucosal dissection over the hemorrhoidal plexus (**b**,**c**). (**d**) This shows the final resection bed. Histopathology revealed a moderately differentiated mucosal carcinoma pT1, G2, L0, V0, and R0, limited to the muscularis mucosae completely removed.

The lesion is first marked circularly at a distance of 3–5 mm with fine coagulation dots using the tip of a diathermic knife (Figure 2a). A lateral submucosal fluid injection is performed in order to lead to a lifting of the lesion and separation of layers. Viscous fluids such as 6% hydroxy ethylic starch (HAES) or 10% glycerol are commonly used to provide a longer lasting fluid cushion than saline solution. This gives a better overview and reduces the risk of perforation during dissection. After a circumferential incision, the lesion is dissected ‘en bloc’ at the submucosal level. Figure 3a–d shows the removal of the findings from Figure 1.

**Figure 4:**
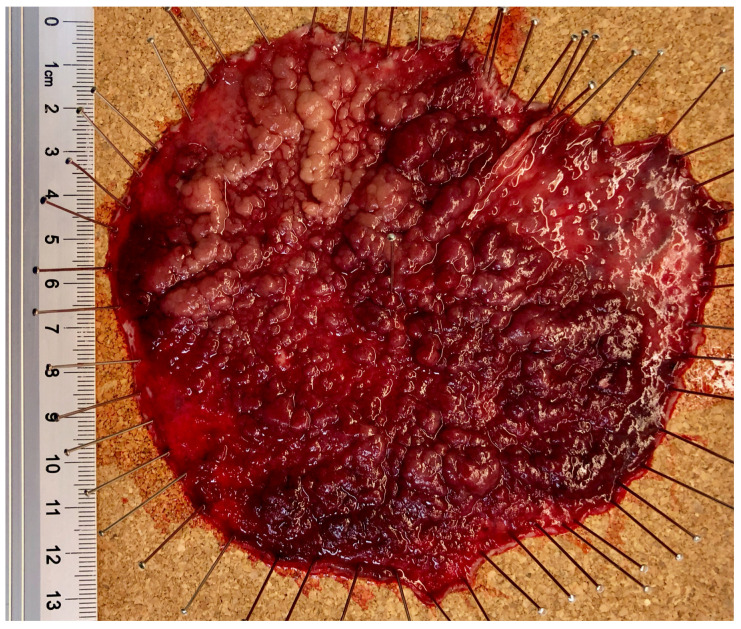
Widespread ESD resection specimen of a recurrent rectal adenoma after prior piecemeal snare resection. Histopathology showed focal high-grade intraepithelial neoplasia (HGIEN) completely resected.

Compared to surgical transanal procedures in the rectum (TEM, TAMIS, etc.), ESD requires less stretching of the anal sphincter. However, the main advantage of ESD is the ability to perform en bloc resection almost regardless of size (Figure 4), with a low local recurrence rate of less than 3% [12]. Organ preservation can be achieved even in the case of mucosal and often submucosally invasive focal carcinomas (see Section 2.3).

The disadvantage of ESD compared to EMR is the increased procedure time, a higher complication rate in terms of bleeding and perforation, and, especially, a long learning curve [26,27,28]. The centralized recording of quality parameters such as the R0 resection rate and relevant complications would be desirable. Fleischmann and colleagues published an analysis of 1000 European cases in 2021 and classified centers according to the frequency of ESDs per year (Table 1) [29]. High-volume centers performed more than 50 ESDs per year, and mid-volume centers 20–50 ESDs. If ≤ 20 ESDs per year were performed, the unit was considered as a ‘low-volume’ ESD unit. En bloc, R0, and curative resection rates were significantly higher in high-volume centers than in medium- or low-volume ESD units. Table 1 details the results. The overall complication rate was 8.3% (83/1000 ESDs analyzed) and was significantly lower in high-volume centers (3%) than in medium-volume centers (12.9%) and low-volume units (10.7%) [29]. At the Vivantes Hospital in Friedrichshain in Berlin, the principal author’s institution until February 2025, the number of ESDs from 2016 to 2024 was 80–100 cases/year. 

### 2.3. Endoscopic Intermuscular Dissection (EID)

Endoscopic intermuscular dissection (EID) is an extension of the ESD technique in the rectum for lesions with a suspicion of deep submucosal invasion (sm2 according to the Kikuchi classification) [30,31]. The technique was first presented by Toyonaga et al. in 2018 for scarred lesions or, in cases of deep submucosal T1/2 infiltration, as “peranal endoscopic myectomy” (PAEM) [32]. In contrast to high-grade displasia or intramucosal cancers with an R0 resection rate of up to 92%, the histologic R0 resection rate drops to 62–64% in the case of deep submucosal infiltration [33,34]. Although surgical transanal excision (TAE; transanal endoscopic microsurgery, TEM) involves all layers, it destroys the embryologic guiding structures, especially in areas of low perirectal fat. If, subsequently, a surgical total mesorectal excision (TME) is required (completion surgery, CS), this leads to a more difficult surgical dissection and reduces the quality of the histologic specimen [35,36]. In contrast, endoscopic intermuscular dissection preserves the outer longitudinal muscle layer and does not touch the submucosal fat layer. In EID, after a generous circumferential marking of 5–10 mm outside the lesion, a fluid cushion such as in ESD is created first and a circumferential incision of the mucosa performed outside the marking dots. Consequently, a small lateral circumferential dissection of the submucosa underneath the mucosa is performed and the muscle layer is exposed. This is followed by a circumferential incision of the inner proper muscular layer starting at the distal and then cranial end, here in inversion. Dissection between the two muscle layers is then performed similar to ESD. This way, the inner circumferential muscle layer is attached to the specimen, while the resection base shows the longitudinal muscle layer at its center.

As a result, the specimen offers to the pathologist a histopathologic examination of the entire submucosal layer. This is especially important in the case of deep submucosal invasion in early rectal cancers without any other risk factors for metastatic spread [37]. In 2022, Moons et al. published the first Dutch multicenter study including 67 patients with a suspicion of T1 cancers with deep submucosal infiltration [30]. They achieved a technical success rate of 96% and a histologic R0 resection rate of 85%. The technique appears very promising, especially for lesions distal to the peritoneal verge. Our first own experiences in eight patients, presented recently, confirmed their results [38,39].

### 2.4. Endoscopic Full-Thickness Resection (eFTR)

**Figure 5:**
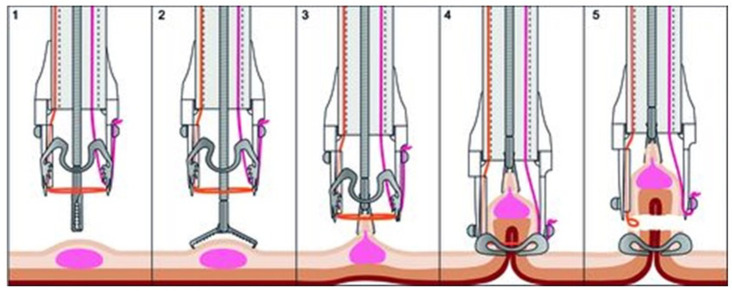
Illustration of the resection procedure using the endoscopic full-thickness resection device (eFTRD; Ovesco, Tuebingen Germany). The system is suited for use in the middle and upper rectum and lesions up to 2 cm in size. After marking, the lesion is grasped with forceps and centered (1,2), then suctioned into a transparent cylindrical attachment cap (3). A special OTSC microclip is then fired (4) and the specimen resected 2–3 mm above the artificial tissue mushroom (5) using an integrated diathermy snare (courtesy of Ovesco, Tübingen, Germany).

Since its launch in late 2015, the Ovesco eFTRD system has expanded the range of endoscopic resection options in the colorectum. Endoscopic full-thickness resection using the full-thickness resection device (eFTRD) removes lesions, including the entire underlying bowel wall (Figure 5) [40,41,42].

**Figure 6:**
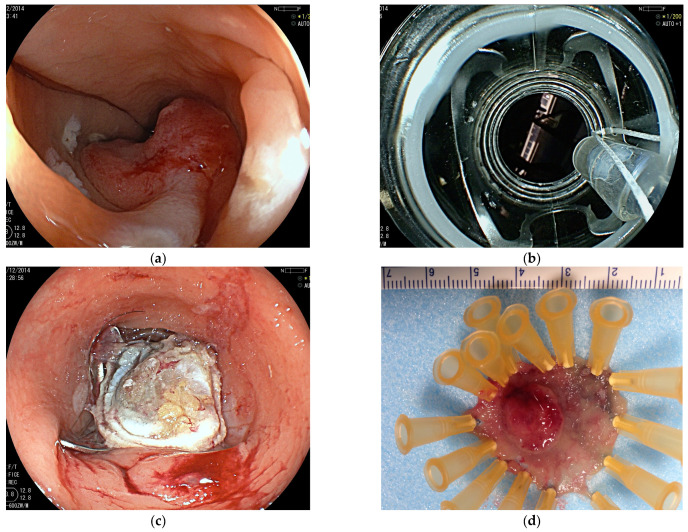
(**a**–**d**) Endoscopic full-thickness resection (eFTR) of an early cancer in the middle rectum (**a**). Endoscopic view with the eFTR device mounted (**b**). Endoscopic image after full-thickness resection with the layers of the rectal wall and central fat (**c**). Specimen pinned after successful resection (**d**).

In addition to suspicious polyps, circumscribed recurrences in scars or adenomatous residuals after piecemeal resection can be removed. The eFTRD system consists of a transparent cap with a clip mounted similar to the OTSC macroclip. A diathermic snare is integrated into the system and sits in a distal inner groove of the cap. The lesion to be resected is first marked with a coagulation probe and then pulled into the transparent cap using forceps plus suction. The special OTSC macroclip is then released. The tissue mushroom inside is now resected 2–3 mm above the nickel–titanium clip by means of the integrated snare and the specimen retrieved. The endoscopic control typically shows all GI wall layers plus central fat inside the OTSC. Figure 6 illustrates a clinical example. Typically, upper- and mid-rectal lesions up to 20 mm are suited for the eFTR system. Küllmer et al. analyzed the results after full-thickness resection in a multicenter retrospective study with 156 T1 colorectal cancers [43]. In the case where the lesion lifted after saline injection in an initial examination, y full-thickness resection was successful in 87.5%. Without a positive lifting sign, the R0 resection rate dropped to 60.9% [43]. Limitations of the use of the FTRD system lie in the distal rectum due to the limited mobility of tissue.

## 3. Special Situation in Early Rectal Cancers

Numerous adenocarcinomas are first diagnosed as suspicious polyps after endoscopic resection, based on a histopathological examination. Colorectal carcinomas whose depth of invasion does not exceed the submucosa are referred to as early carcinomas. These include, on the one hand, so-called mucosal or intraepithelial carcinomas (pTis) and pT1 carcinomas with deep infiltration into the submucosa. Early carcinomas differ considerably in their prognosis depending on the risk profile. In principle, it should be noted that, for carcinomas in the pT1 stage, local ‘en bloc’ resection is sufficient as the sole therapeutic measure if the following conditions for classification in a low-risk situation are met [12,37,44]:

- Diameter < 3 cm;
- G1/2: good or moderate histological differentiation;
- L0: no infiltration of lymph vessels;
- V0: no infiltration of blood vessels;
- R0: complete resection;
- No “scattered” tumor cells at the invasion front of the carcinoma (“budding”).

If, on the other hand, risk factors are present, such as a poor degree of differentiation, lymph vessel infiltration, tumor-positive resection margins, or tumor budding, it is referred to as a high-risk T1 carcinoma. According to recent studies, deep submucosal invasion (>1000 µm) as the only risk factor without further negative predictive factors is only a minor risk factor for metastasis [37]. Currently, endoscopic resection techniques concentrate on T1a early cancers. According to Chen et al., the risk of lymph node metastasis in high-risk T1 rectal cancer is 20.7% (with one risk factor) and up to 36.4% with several risk factors [45]. The most important risk factors for lymph node meta-metastasis are lymphatic vessel invasion (L1) and poor differentiation [15,37]. The above parameters usually result in an indication for secondary surgical–oncological resection. Classically, this should be performed within 30 days without adverse effects on the oncological outcome [36,46].

## 4. Conclusions

Today, endoscopic procedures allow large resections of adenomas and the curative resection of low-risk T1 carcinomas in the rectum. Endoscopic mucosal resection (EMR) using a snare and piecemeal resection after submucosal saline injection can be an adequate procedure for flat, non-polypoid lesions with a low risk of malignancy [7]. It seems, today, inadequate for polypoid lesions, which cannot be removed in one piece due to the increased risk of focal malignancy [7]. Endoscopic submucosal dissection (ESD) has led to a significant reduction in local recurrence rates compared to piecemeal resection for widespread adenomas. This is particularly true for lesions in the distal rectum and the anorectal junction, where ESD should be the preferred approach. Endoscopic intermuscular dissection (EID) represents a new perspective for suspicious lesions and proven early rectal cancers as it provides the entire submucosal layer for histopathological examination. Further results have to be awaited. Endoscopic resection seems to be the first step to be recommended in the case of endoscopically feasible resection. Takamaru et al. found no oncological disadvantage for primary ESD and a necessary secondary surgical resection compared to primary surgical resection in over 800 patients with high-risk colorectal cancer [47]. Primary endoscopic resection for high-risk lesions at expert centers with a high frequency in ESD and EID should, therefore, always be the first step.

## 5. Note

This article is, in part, based on the manuscript‚ “Endoskopische Resektionsverfahren von flächigen Präkanzerosen und Frühkarzinomen im Rektum”, published recently by the authors as an open access article in German [24].

## Figures and Tables

**Table 1:** Volume-dependent quality of ESD outcomes after Fleischmann et al., 2021 [29].

Volume	*n* ESDs/Year	“En Bloc” Resection Rate	R0 Resection Rate	Curative Resection Rate	Major Complications
**High-Volume ESD Centers**	>50	96.0%	87.9%	82.9%	3.00%
**Mid-Size ESD Centers**	>20–50	93.9%	77.3%	69.8%	12.90%
**Low-Volume ESD Units**	≤20	86.3%	68.5%	61.0%	10.70%
